# Supplementary material for: Understanding early HIV-1 rebound dynamics following antiretroviral therapy interruption: The importance of effector cell expansion
Source: PLoS Pathog. 2024 Jul 29;20(7):e1012236. doi: 10.1371/journal.ppat.1012236 (PMC11309407; doi:10.1371/journal.ppat.1012236)
Supplement: S1 Text — Table A. Model fit comparison (Monolix 2023R1, Lixoft, SA, Antony, France). There are four categories separated by double-lines. Bolded values are the lowest values in the category. The first category contains the Conway and Perelson model with five variations without covariates. The second category tests single covariates for the best fit model in the first category. The third category shows two examples of using two covariates that produce the lowest fitting error. The last category tests the Simplified Model 1 without random effect for KB. Without a random effect, the population estimate for KB is 16.88 (S.E. 0.12)cells mL-1. Table B. Best-fit population parameters for the Conway & Perelson Model vs. reference values from Conway and Perelson [49]. Note the estimated values of KD and d result in a negligible effect of exhaustion. Table C. Best-fit population parameters for the Simplified Model 2 vs. reference values from Conway and Perelson [49]. Table D. Best-fit population parameters for the Simplified Model 3 vs. reference values from Conway and Perelson [49]. Note that the estimated values of KD and d together imply the exhaustion effect is very small, which may be because of this particular set of participants or that it cannot be observed from the limited data. Table E. Best-fit population parameters for the Simplified Model 4 vs. reference values from Conway and Perelson [49]. Note that m* is not the same as m. Table F. Best-fit population parameters for the Simplified Model 5 vs. reference values from Conway and Perelson [49]. Note that m* is not the same as m. Table G. Individual best-fit parameters–Conway & Perelson model. Table H. Individual best-fit parameters–Simplified Model 1. Table I. Individual best-fit parameters–Simplified Model 1 with a covariate on KB (1 is PTC and 2 is NC). For KB, the mean and SD (%) are reported for individual group PTC/NC. Table J. Individual best-fit parameters–Simplified Model 2. Table K. Individual best-fit parameters–S [file ppat.1012236.s001.docx]

**S1 Text. Supplementary Materials.**

Understanding early HIV-1 rebound dynamics following antiretroviral therapy interruption: The importance of effector cell expansion.

**Section A. Equations for each model**

Here, we present the equations for all six models that we fit to the viral load data for the 24 PWH that we studied in the main text. Note that $V=\frac{p}{c}I$ in all models.

Conway & Perelson model

$$T^{'}=\lambda_{T}-d_{T}T-\beta VT$$

$$I^{'}=\left( 1-f_{L} \right)\beta VT+aL-\delta I-mEI$$

$$L^{'}=f_{L}\beta VT-d_{L}L-aL+\rho L$$

$$E^{'}=\lambda_{E}+\frac{bEI}{K_{B}+I}-\frac{dEI}{K_{D}+I}-d_{E}E.$$

Simplified Model 1 (as above, but$d=0$)

$$T^{'}=\lambda_{T}-d_{T}T-\beta VT$$

$$I^{'}=\left( 1-f_{L} \right)\beta VT+aL-\delta I-mEI$$

$$L^{'}=f_{L}\beta VT-d_{L}L-aL+\rho L$$

$$E^{'}=\lambda_{E}+\frac{bEI}{K_{B}+I}-d_{E}E.$$

Simplified Model 2 (as Simplified Model 1, but no dynamics of latent cells$L^{'}\equiv0$)

$$T^{'}=\lambda_{T}-d_{T}T-\beta VT$$

$$I^{'}=\left( 1-f_{L} \right)\beta VT+aL_{0}-\delta I-mEI$$

$$E^{'}=\lambda_{E}+\frac{bEI}{K_{B}+I}-d_{E}E.$$

Simplified Model 3 (as Simplified Model 2, but$d\neq0$, so with exhaustion of effector cells)

$$T^{'}=\lambda_{T}-d_{T}T-\beta VT$$

$$I^{'}=\left( 1-f_{L} \right)\beta VT+aL_{0}-\delta I-mEI$$

$$E^{'}=\lambda_{E}+\frac{bEI}{K_{B}+I}-\frac{dEI}{K_{D}+I}-d_{E}E.$$

Simplified Model 4 (as Simplified Model 2, but no explicit dynamics of effector cells$E^{'}\equiv0$)

$$T^{'}=\lambda_{T}-d_{T}T-\beta VT$$

$$I^{'}=\left( 1-f_{L} \right)\beta VT+aL_{0}-\delta I-m_{f}I.$$

Simplified Model 5 (as Simplified Model 1, but no explicit dynamics of effector cells$E^{'}\equiv0$)

$$T^{'}=\lambda_{T}-d_{T}T-\beta VT$$

$$I^{'}=\left( 1-f_{L} \right)\beta VT+aL-\delta I-m_{f}I$$

$$L^{'}=f_{L}\beta VT-d_{L}L-aL+\rho L.$$

**Section B. Model fits**

Table A shows the comparisons of models under different assumptions. Figs A-F (and Fig 1, main text) are the best fit of each model, with the population estimates provided in Tables B-F. Figs G-J show the stratifications of best fit parameters for the Conway & Perelson model, Simplified Models 1, 2 and 3. Tables G-M give the individual estimates for the best fit of the Conway & Perelson model and Simplified Models 1 (with and without covariate on$K_{B}$) through 5.

| Model | Effector cell exhaustion | Dynamic latent reservoir | Dynamic effector cells | -2LL | | BICc | | Figure  (Best-fit) | |  |
| --- | --- | --- | --- | --- | --- | --- | --- | --- | --- | --- |
| Conway & Perelson | Yes | Yes | Yes | 547.38 | | 625.29 | | A | |  |
| Simplified Model 1 | No | Yes | Yes | **545.33** | | **605.22** | | 1 (main) | |  |
| Simplified Model 2 | No | No | Yes | 548.12 | | 608.02 | | C | |  |
| Simplified Model 3 | Yes | No | Yes | 545.66 | | 623.57 | | D | |  |
| Simplified Model 4 |  | No | No | 894.15 | | 927.01 | | E | |  |
| Simplified Model 5 |  | Yes | No | 893.17 | | 926.04 | | F | |  |
| Simplified Model 1 (covariate on $K_{B}$) | | | | | **515.74** | | **578.81** | | B | |
| Simplified Model 1 (covariate on $\beta$) | | | | | 547.94 | | 611.01 | | — | |
| Simplified Model 1 (covariate on $\lambda_{E}$) | | | | | 546.29 | | 609.36 | | — | |
| Simplified Model 1 (covariate on $b$) | | | | | 532.59 | | 595.66 | | — | |
| Simplified Model 1 (covariate on $p$) | | | | | 546.98 | | 610.05 | | — | |
| Simplified Model 1 (covariate on $m$) | | | | | 543.33 | | 606.40 | | — | |
| Simplified Model 1 (covariates on $K_{B}$ and $m$) | | | | | **513.58** | | **579.83** | | **—** | |
| Simplified Model 1 (covariates on $K_{B}$ and $b$) | | | | | 514.84 | | 581.09 | | — | |
| Simplified Model 1 (without random effect for $K_{B}$) | | | | | 593.83 | | 650.55 | | — | |

Table A. Model fit comparison (Monolix 2023R1, Lixoft, SA, Antony, France). There are four categories separated by double-lines. Bolded values are the lowest values in the category. The first category contains the Conway and Perelson model with five variations without covariates. The second category tests single covariates for the best fit model in the first category. The third category shows two examples of using two covariates that produce the lowest fitting error. The last category tests the Simplified Model 1 without random effect for$K_{B}$. Without a random effect, the population estimate for $K_{B}$ is 16.88 (S.E. 0.12)$cells mL^{-1}$.


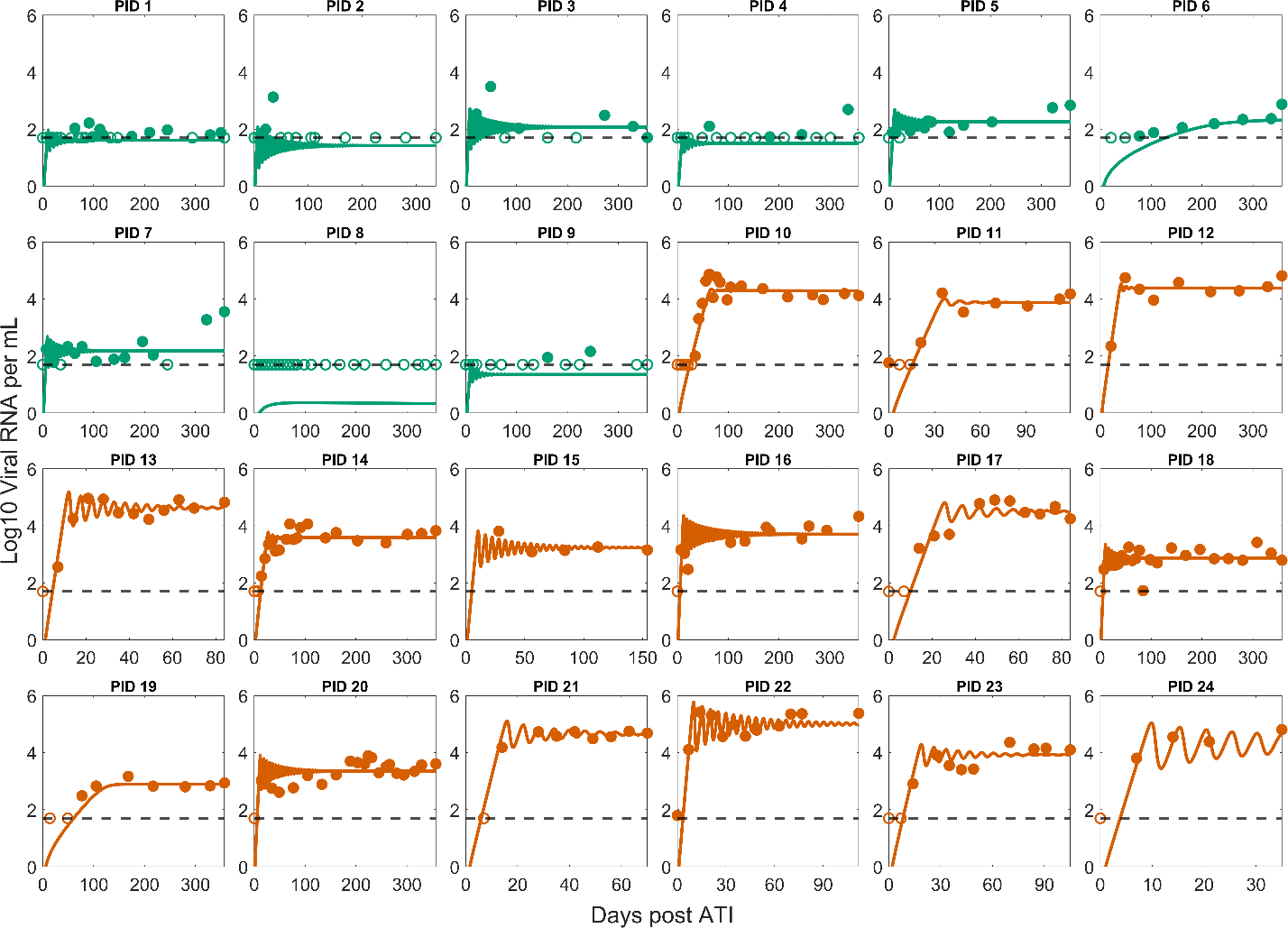


Fig A. Best fit of the Conway & Perelson model to the post-ATI data from Sharaf et al. [57]. Green indicates PTC. Dark orange indicates NC. The horizontal dashed line is the limit of detection. Open circles are data points below the limit of detection (50 viral RNA copies/mL). Filled circles are data points above the limit of detection.


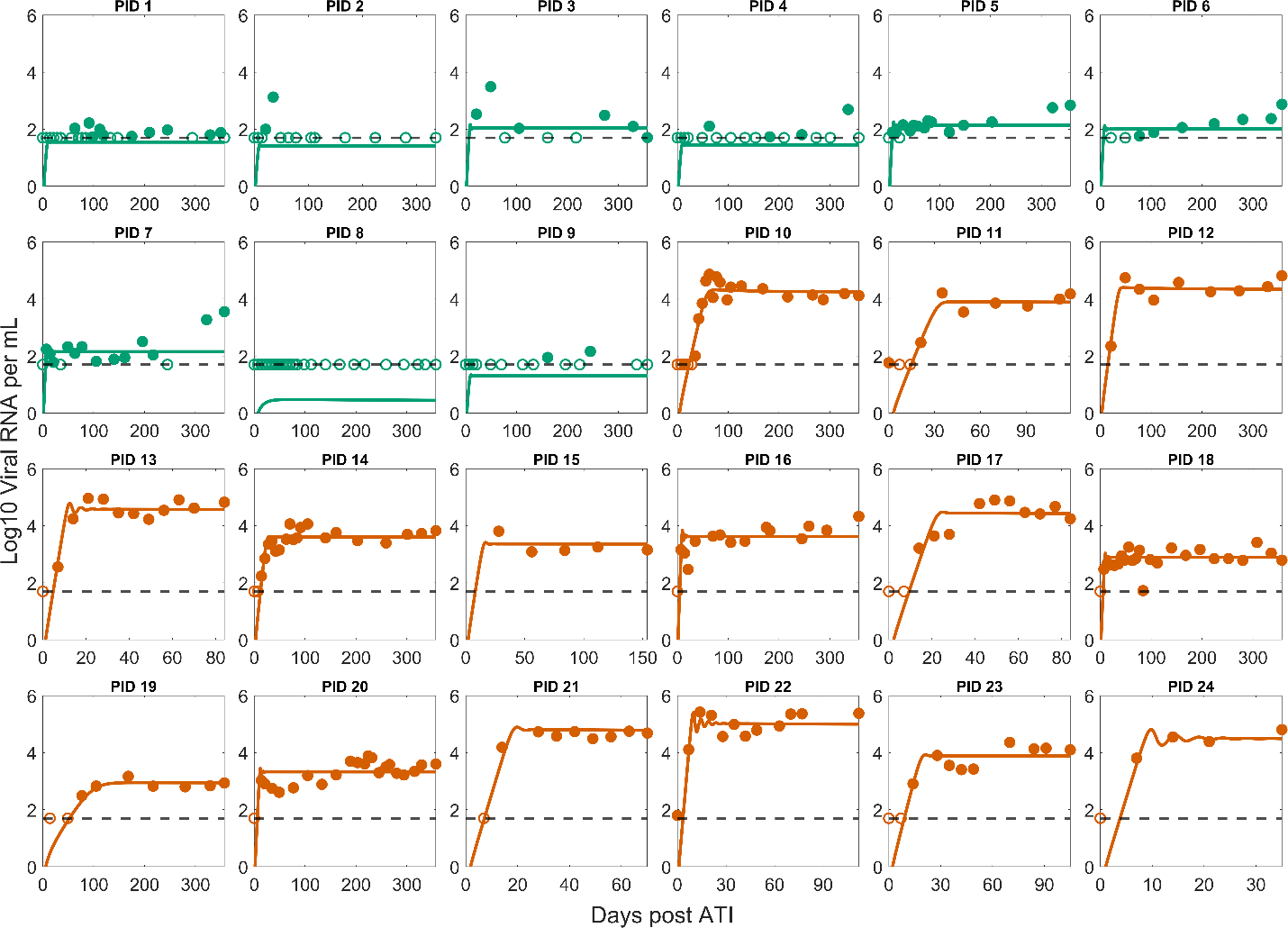


Fig B. Best fit of Simplified Model 1 with a covariate on$K_{B}$ to the post-ATI data from Sharaf et al. [57]. Lines show the model predictions using the best fit parameter values from Simplified Model 1 with a covariate on$K_{B}$. Green indicates PTC. Dark orange indicates NC. The horizontal dashed line is the limit of detection (50 viral RNA copies/mL). Open circles are data points below the limit of detection. Filled circles are data points above the limit of detection.


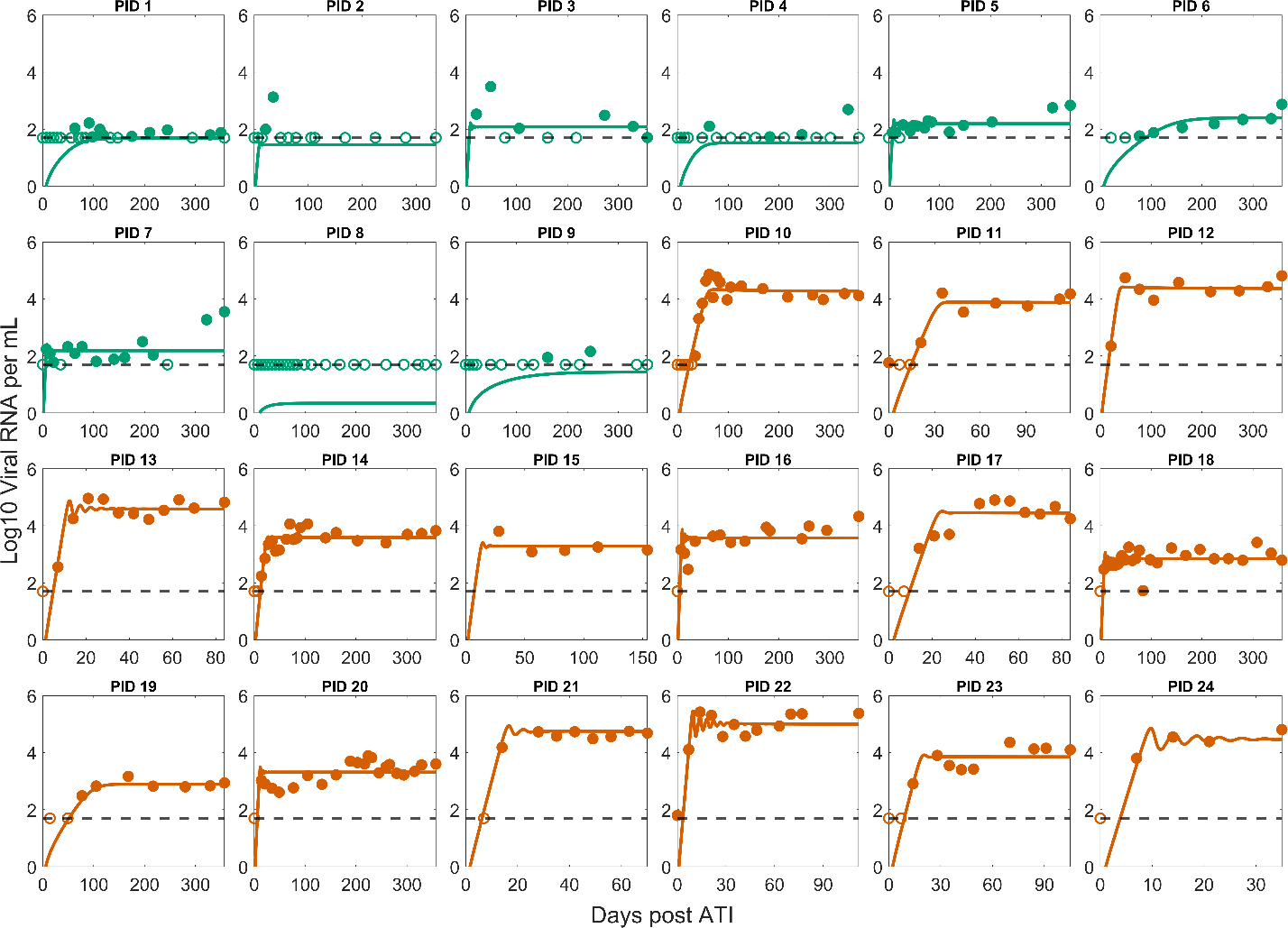


Fig C. Best fit of the Simplified Model 2 to the post-ATI data from Sharaf et al. [57]. Green indicates PTC. Dark orange indicates NC. The horizontal dashed line is the limit of detection. Open circles are data points below the limit of detection (50 viral RNA copies/mL). Filled circles are data points above the limit of detection.


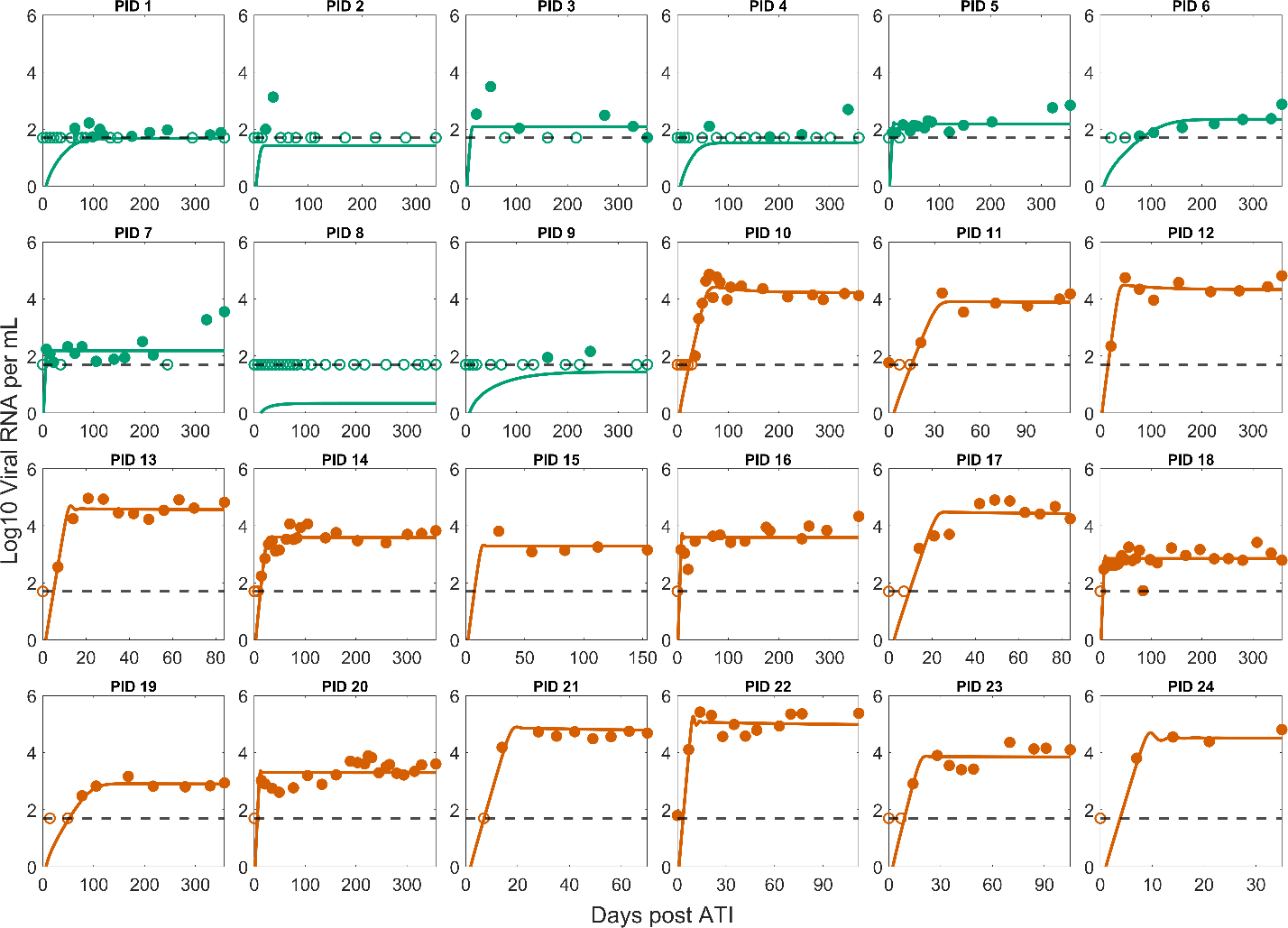


Fig D. Best fit of the Simplified Model 3 to the post-ATI data from Sharaf et al. [57]. Green indicates PTC. Dark orange indicates NC. The horizontal dashed line is the limit of detection. Open circles are data points below the limit of detection (50 viral RNA copies/mL). Filled circles are data points above the limit of detection.


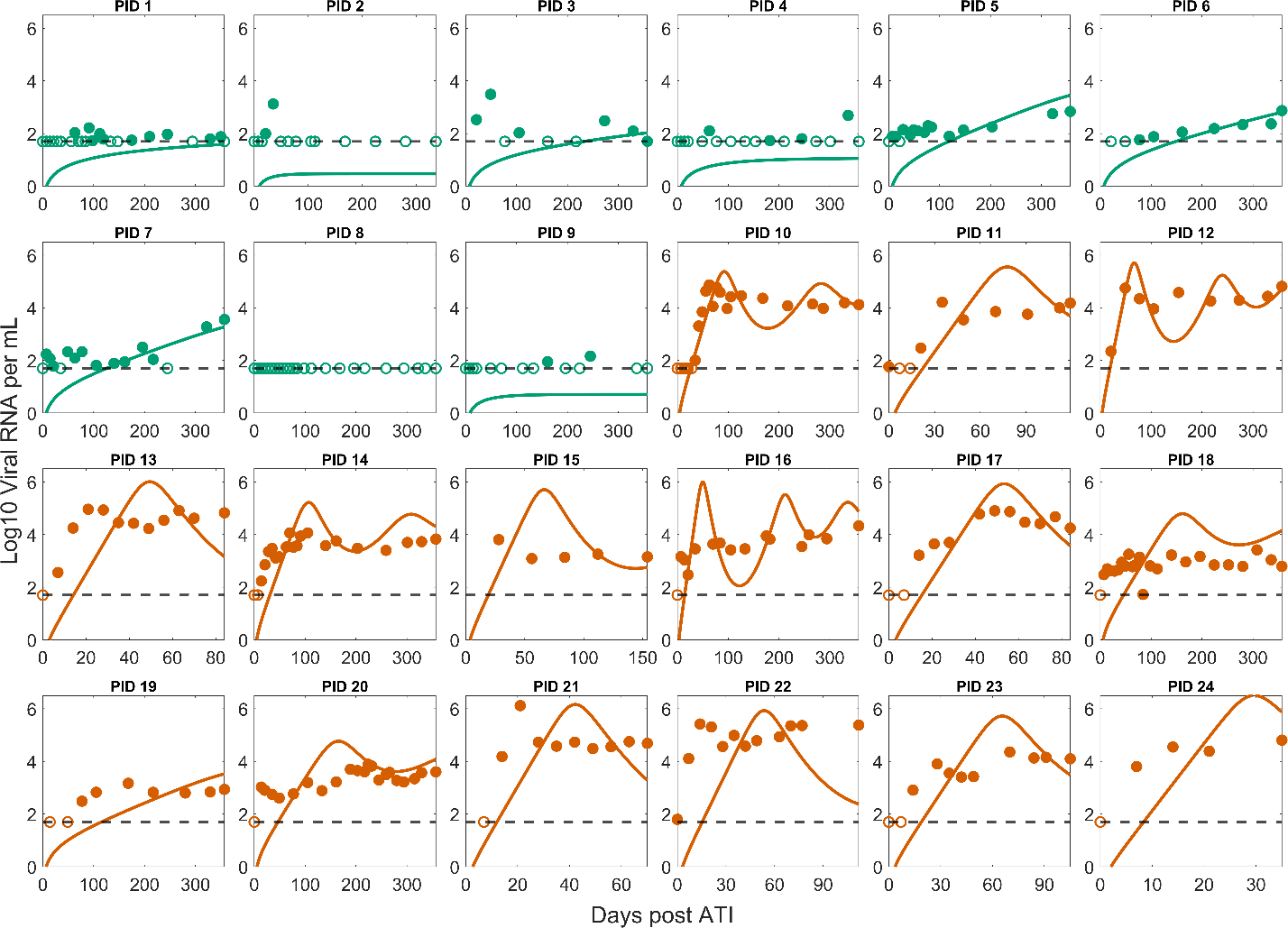


Fig E. Best fit of the Simplified Model 4 to the post-ATI data from Sharaf et al. [57]. Green indicates PTC. Dark orange indicates NC. The horizontal dashed line is the limit of detection. Open circles are data points below the limit of detection (50 viral RNA copies/mL). Filled circles are data points above the limit of detection.


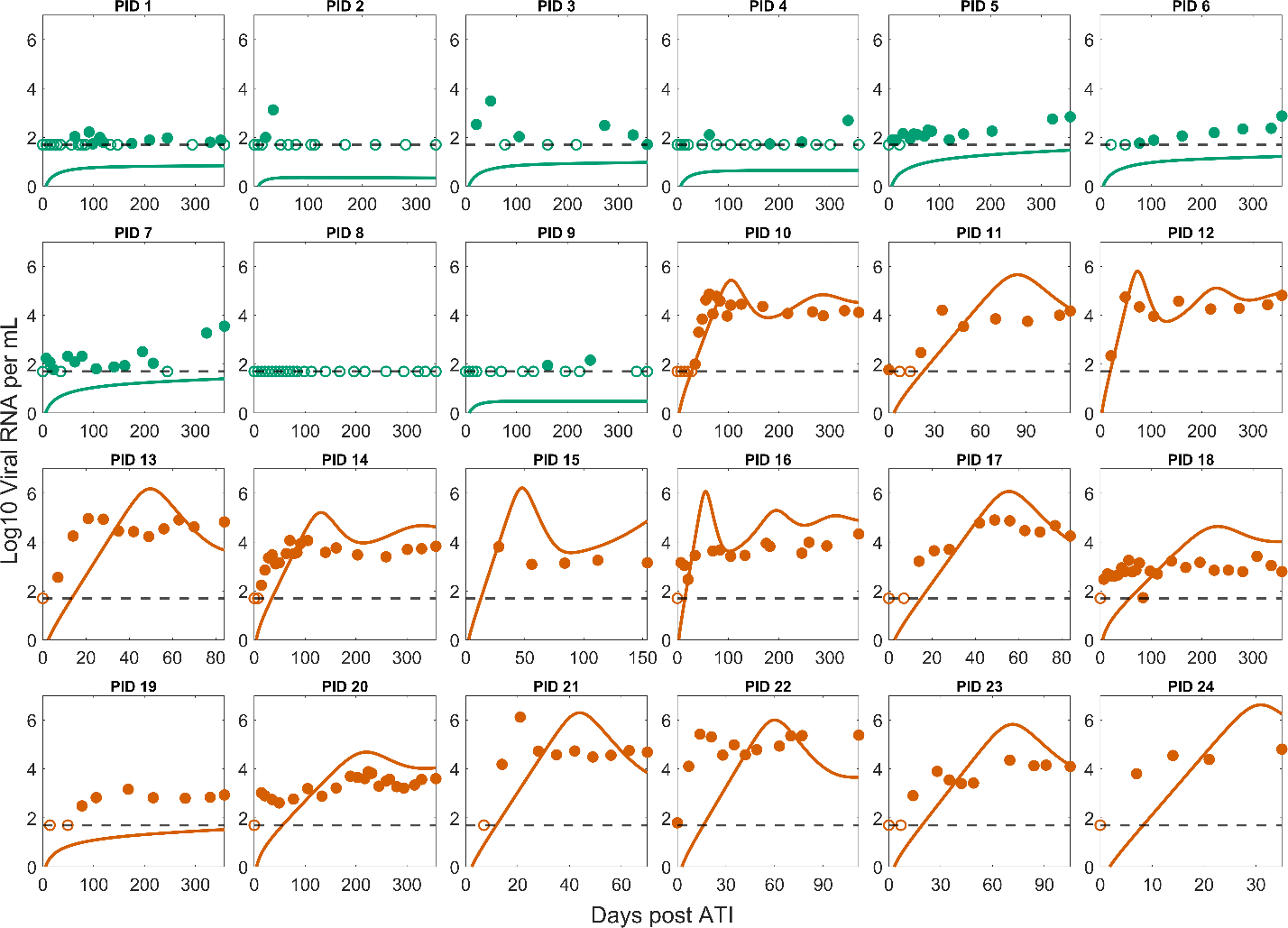


Fig F. Best fit of the Simplified Model 5 to the post-ATI data from Sharaf et al. [57]. Green indicates PTC. Dark orange indicates NC. The horizontal dashed line is the limit of detection. Open circles are data points below the limit of detection (50 viral RNA copies/mL). Filled circles are data points above the limit of detection.

**Section C. Stratification of best fit parameters**


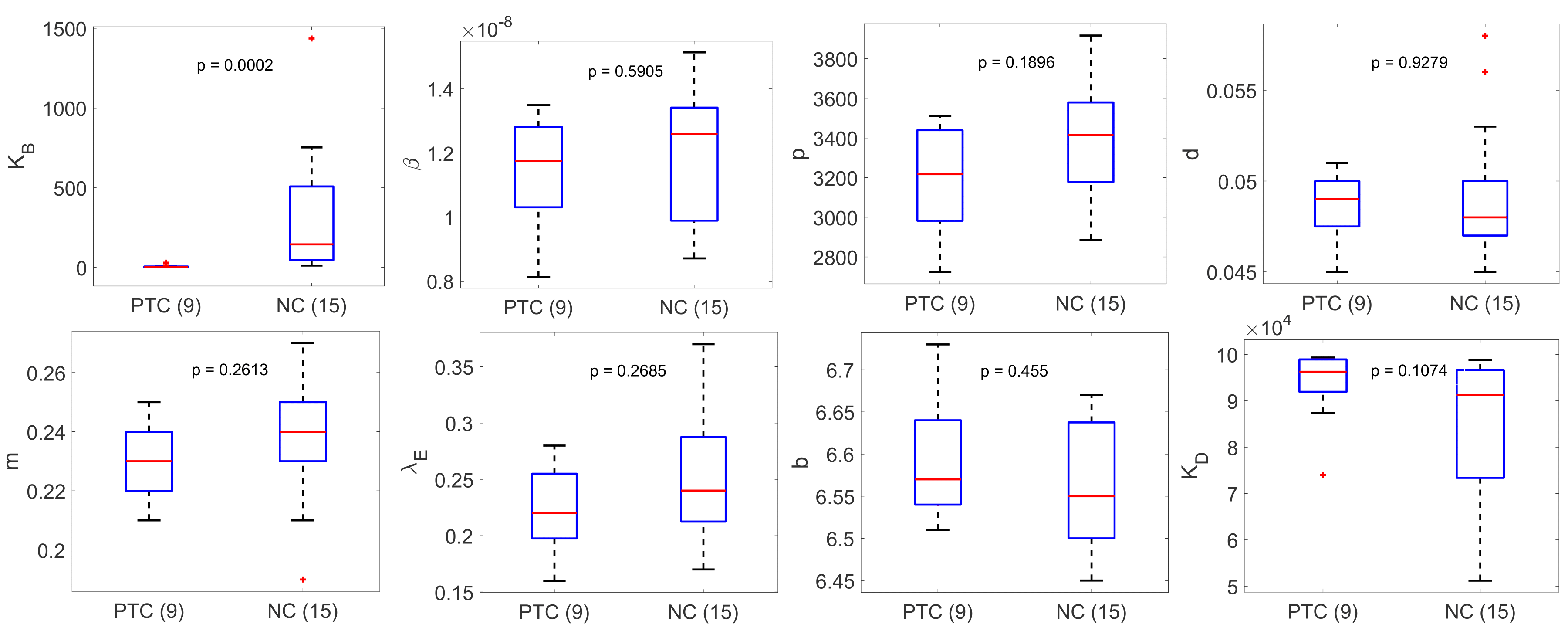


Fig G. Summary of best-fit parameters in the Conway & Perelson Model stratified based on PTC or NC. The difference in the values of $K_{B}$ is the most significant between PTC and NC.


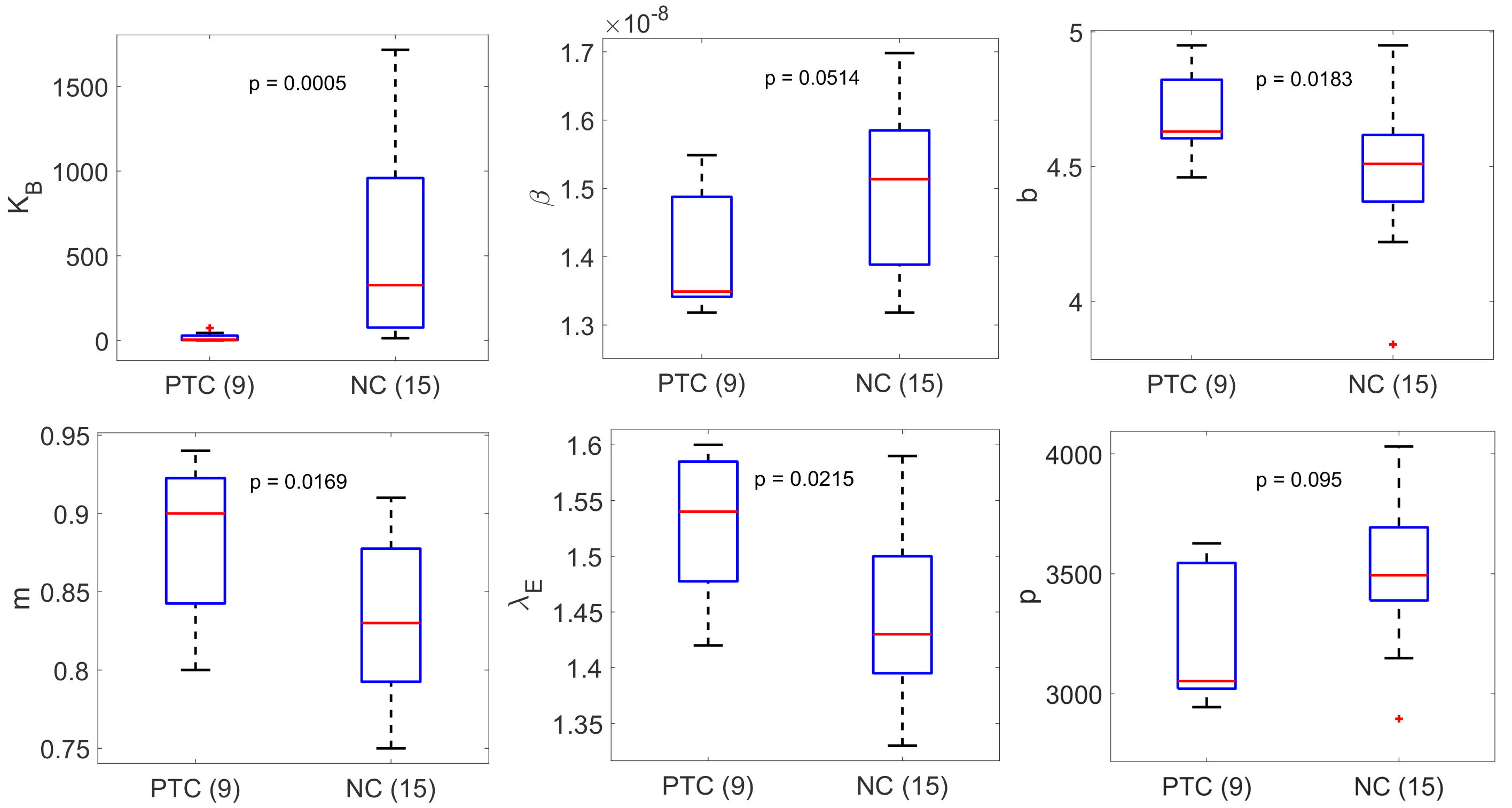


Fig H. Summary of best fit parameters in Simplified Model 1 stratified based on PTC or NC. The difference in the values of $K_{B}$ is the most significant between PTC and NC.


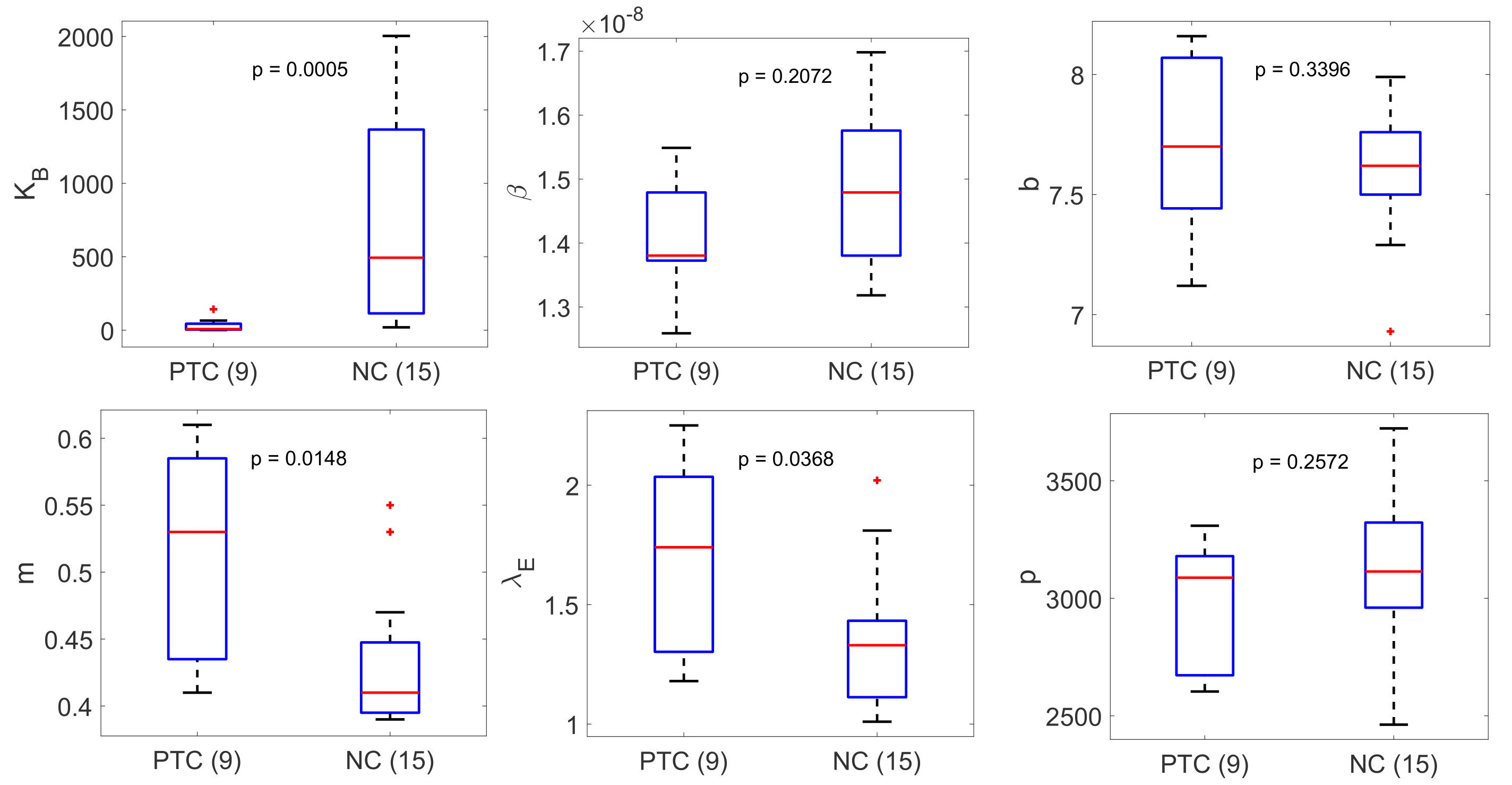


Fig I. Summary of best-fit parameters in the Simplified Model 2 stratified based on PTC or NC. The difference in the values of $K_{B}$ is the most significant between PTC and NC.


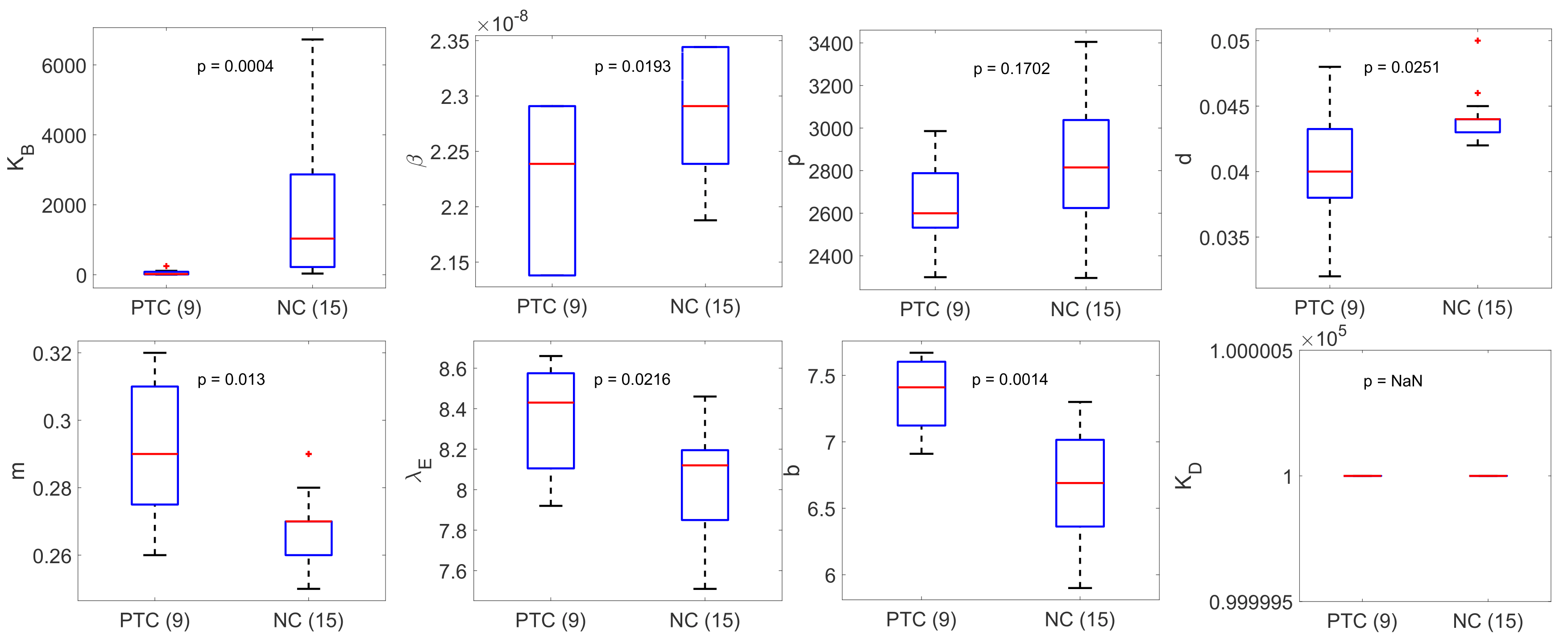


Fig J. Summary of best-fit parameters in the Simplified Model 3 stratified based on PTC or NC. The difference in the values of $K_{B}$ is the most significant between PTC and NC.

**Section D. Best fit parameters for each individual**

| Parameter | Best fit values (population estimate) | Reference values |
| --- | --- | --- |
| $\beta$ | $1.20\times{10}^{-8}$  $mL \left( HIV RNA copies \right)^{-1}day^{-1}$ | $1.5\times{10}^{-8}$  $mL \left( HIV RNA copies \right)^{-1}day^{-1}$ |
| $\lambda_{E}$ | $0.25 cells mL^{-1}day^{-1}$ | $1 cells mL^{-1}day^{-1}$ |
| $m$ | $0.24 mL cells^{-1}day^{-1}$ | $0.42 mL cells^{-1}day^{-1}$ |
| $b$ | $6.57 day^{-1}$ | $1 day^{-1}$ |
| $K_{B}$ | $29.35 cells mL^{-1}$ | $0.1 cells mL^{-1}$ |
| $p$ | $3337 \left( HIV RNA copies \right) day^{-1}$ | $2000 \left( HIV RNA copies \right) day^{-1}$ |
| $K_{D}$ | $95188 cells mL^{-1}$ | $5 cells mL^{-1}$ |
| $d$ | $0.05 day^{-1}$ | $2 {day}^{-1}$ |

Table B. Best-fit population parameters for the Conway & Perelson Model vs. reference values from Conway and Perelson (49). Note the estimated values of $K_{D}$ and $d$ result in a negligible effect of exhaustion.

| Parameter | Best fit values (population estimate) | Reference values |
| --- | --- | --- |
| $\beta$ | $1.51\times{10}^{-8}$  $mL\left( HIV RNA copies \right)^{-1} day^{-1}$ | $1.5\times{10}^{-8}$  $mL \left( HIV RNA copies \right)^{-1}day^{-1}$ |
| $\lambda_{E}$ | $1.33 cells mL^{-1}day^{-1}$ | $1 cells mL^{-1}day^{-1}$ |
| $m$ | $0.44 mL cells^{-1}day^{-1}$ | $0.42 mL cells^{-1}day^{-1}$ |
| $b$ | $7.66 day^{-1}$ | $1 day^{-1}$ |
| $K_{B}$ | $65.99 cells mL^{-1}$ | $0.1 cells mL^{-1}$ |
| $p$ | $3104 \left( HIV RNA copies \right) day^{-1}$ | $2000 \left( HIV RNA copies \right) day^{-1}$ |

Table C. Best-fit population parameters for the Simplified Model 2 vs. reference values from Conway and Perelson (49).

| Parameter | Best fit values (population estimate) | Reference values |
| --- | --- | --- |
| $\beta$ | $2.29\times{10}^{-8}$  $mL \left( HIV RNA copies \right)^{-1}day^{-1}$ | $1.5\times{10}^{-8}$  $mL \left( HIV RNA copies \right)^{-1}day^{-1}$ |
| $\lambda_{E}$ | $8.06 cells mL^{-1}day^{-1}$ | $1 cells mL^{-1}day^{-1}$ |
| $m$ | $0.27 mL cells^{-1}day^{-1}$ | $0.42 mL cells^{-1}day^{-1}$ |
| $b$ | $6.8 day^{-1}$ | $1 day^{-1}$ |
| $K_{B}$ | $122.97 cells mL^{-1}$ | $0.1 cells mL^{-1}$ |
| $p$ | $2798 \left( HIV RNA copies \right) day^{-1}$ | $2000 \left( HIV RNA copies \right) day^{-1}$ |
| $K_{D}$ | *100000* $cells mL^{-1}$ | $5 cells mL^{-1}$ |
| $d$ | *0.044* $day^{-1}$ | $2 day^{-1}$ |

Table D. Best-fit population parameters for the Simplified Model 3 vs. reference values from Conway and Perelson [49]. Note that the estimated values of $K_{D}$ and $d$ together imply the exhaustion effect is very small, which may be because of this particular set of participants or that it cannot be observed from the limited data.

| Parameter | Best fit values (population estimate) | Reference values |
| --- | --- | --- |
| $\beta$ | $2.04\times{10}^{-8}$  $mL \left( HIV RNA copies \right)^{-1}day^{-1}$ | $1.5\times{10}^{-8}$  $mL \left( HIV RNA copies \right)^{-1}day^{-1}$ |
| $m^{*}$ | $1.4 mL day^{-1}$ |  |
| $p$ | $2855 \left( HIV RNA copies \right) day^{-1}$ | $2000 \left( HIV RNA copies \right) day^{-1}$ |

Table E. Best-fit population parameters for the Simplified Model 4 vs. reference values from Conway and Perelson [49]. Note that $m^{*}$ is not the same as$m$.

| Parameter | Best fit values (population estimate) | Reference values |
| --- | --- | --- |
| $\beta$ | $1.48\times{10}^{-8}$  $mL \left( HIV RNA copies \right)^{-1}day^{-1}$ | $1.5\times{10}^{-8}$  $mL \left( HIV RNA copies \right)^{-1}day^{-1}$ |
| $m^{*}$ | $1.18 mL day^{-1}$ |  |
| $p$ | $3608 \left( HIV RNA copies \right) day^{-1}$ | $2000 \left( HIV RNA copies \right) day^{-1}$ |

Table F. Best-fit population parameters for the Simplified Model 5 vs. reference values from Conway and Perelson [49]. Note that $m^{*}$ is not the same as$m$.

| **ID** | $\boldsymbol{-lo}\boldsymbol{g}_{\boldsymbol{10}}\left( \boldsymbol{\beta} \right)$ | $\boldsymbol{\lambda}_{\boldsymbol{E}}$ | $\boldsymbol{b}$ | $\boldsymbol{K}_{\boldsymbol{B}}$ | $\boldsymbol{m}$ | $\boldsymbol{d}$ | $\boldsymbol{K}_{\boldsymbol{D}}$ | $\boldsymbol{p}$ |
| --- | --- | --- | --- | --- | --- | --- | --- | --- |
| 1 | 7.9 | 0.21 | 6.51 | 0.77 | 0.22 | 0.046 | 74015 | 2935 |
| 2 | 7.87 | 0.19 | 6.61 | 0.41 | 0.21 | 0.049 | 96258 | 3510 |
| 3 | 7.87 | 0.16 | 6.57 | 1.86 | 0.22 | 0.048 | 98839 | 3427 |
| 4 | 7.93 | 0.22 | 6.73 | 0.6 | 0.24 | 0.049 | 99161 | 3098 |
| 5 | 7.93 | 0.2 | 6.55 | 3 | 0.22 | 0.045 | 87383 | 3334 |
| 6 | 8.09 | 0.27 | 6.51 | 13.46 | 0.24 | 0.048 | 97834 | 2998 |
| 7 | 7.9 | 0.25 | 6.59 | 2.41 | 0.23 | 0.05 | 93462 | 3478 |
| 8 | 8.08 | 0.28 | 6.57 | 29.73 | 0.25 | 0.05 | 95571 | 2723 |
| 9 | 7.96 | 0.25 | 6.73 | 0.41 | 0.24 | 0.051 | 99333 | 3217 |
| 10 | 8.06 | 0.23 | 6.49 | 410.9 | 0.24 | 0.058 | 96410 | 3136 |
| 11 | 8.05 | 0.23 | 6.5 | 131.1 | 0.23 | 0.05 | 91311 | 3416 |
| 12 | 8.01 | 0.22 | 6.55 | 470.7 | 0.23 | 0.05 | 71299 | 3095 |
| 13 | 7.85 | 0.37 | 6.67 | 680.2 | 0.26 | 0.048 | 93935 | 3592 |
| 14 | 7.99 | 0.25 | 6.64 | 72.74 | 0.24 | 0.046 | 98802 | 3168 |
| 15 | 7.87 | 0.24 | 6.5 | 26.73 | 0.23 | 0.047 | 97931 | 3461 |
| 16 | 7.9 | 0.17 | 6.53 | 73.98 | 0.19 | 0.045 | 98378 | 3659 |
| 17 | 7.97 | 0.17 | 6.52 | 520.2 | 0.21 | 0.05 | 71515 | 3208 |
| 18 | 7.89 | 0.32 | 6.64 | 11.83 | 0.26 | 0.047 | 92294 | 3542 |
| 19 | 8.06 | 0.28 | 6.48 | 27.61 | 0.25 | 0.048 | 90667 | 2886 |
| 20 | 7.88 | 0.2 | 6.67 | 36.46 | 0.22 | 0.047 | 88941 | 3423 |
| 21 | 7.88 | 0.29 | 6.45 | 752.7 | 0.27 | 0.053 | 96695 | 3328 |
| 22 | 7.82 | 0.32 | 6.61 | 1436 | 0.24 | 0.045 | 68560 | 3917 |
| 23 | 7.94 | 0.21 | 6.63 | 144.5 | 0.24 | 0.05 | 79011 | 3286 |
| 24 | 7.82 | 0.28 | 6.61 | 317.3 | 0.25 | 0.056 | 51197 | 3670 |
| Mean | 7.94 | 0.24 | 6.58 | 215.2 | 0.23 | 0.049 | 88700 | 3313 |
| SD (%) | 1.02 | 21.13 | 1.16 | 158.5 | 7.58 | 6.43 | 14.02 | 8.28 |

Table G. Individual best-fit parameters – Conway & Perelson model.

| **ID** | $\boldsymbol{-lo}\boldsymbol{g}_{\boldsymbol{10}}\left( \boldsymbol{\beta} \right)$ | $\boldsymbol{\lambda}_{\boldsymbol{E}}$ | $\boldsymbol{b}$ | $\boldsymbol{K}_{\boldsymbol{B}}$ | $\boldsymbol{m}$ | $\boldsymbol{p}$ |
| --- | --- | --- | --- | --- | --- | --- |
| 1 | 7.87 | 1.6 | 4.63 | 14.67 | 0.92 | 3053 |
| 2 | 7.85 | 1.49 | 4.95 | 0.99 | 0.9 | 3468 |
| 3 | 7.83 | 1.49 | 4.78 | 2.95 | 0.85 | 3536 |
| 4 | 7.87 | 1.58 | 4.61 | 5.08 | 0.91 | 3053 |
| 5 | 7.82 | 1.44 | 4.95 | 3.33 | 0.8 | 3570 |
| 6 | 7.88 | 1.56 | 4.46 | 73.62 | 0.89 | 3040 |
| 7 | 7.81 | 1.42 | 4.68 | 2.92 | 0.82 | 3627 |
| 8 | 7.88 | 1.6 | 4.59 | 45.02 | 0.94 | 2964 |
| 9 | 7.87 | 1.54 | 4.62 | 24.08 | 0.93 | 2945 |
| 10 | 7.85 | 1.43 | 4.67 | 1716 | 0.87 | 2896 |
| 11 | 7.87 | 1.55 | 4.22 | 326.9 | 0.89 | 3422 |
| 12 | 7.86 | 1.51 | 4.23 | 1222 | 0.88 | 3225 |
| 13 | 7.8 | 1.38 | 4.43 | 568.4 | 0.78 | 3707 |
| 14 | 7.86 | 1.54 | 4.63 | 149 | 0.89 | 3463 |
| 15 | 7.82 | 1.46 | 4.58 | 42.88 | 0.83 | 3494 |
| 16 | 7.79 | 1.39 | 4.35 | 52.61 | 0.79 | 3787 |
| 17 | 7.84 | 1.47 | 4.43 | 795.3 | 0.84 | 3379 |
| 18 | 7.8 | 1.41 | 4.95 | 13.41 | 0.8 | 3651 |
| 19 | 7.88 | 1.59 | 4.64 | 161.2 | 0.91 | 3148 |
| 20 | 7.81 | 1.41 | 4.51 | 34.97 | 0.81 | 3650 |
| 21 | 7.82 | 1.43 | 4.52 | 1422 | 0.81 | 3494 |
| 22 | 7.77 | 1.33 | 3.84 | 1014 | 0.75 | 4031 |
| 23 | 7.84 | 1.47 | 4.51 | 191.5 | 0.84 | 3418 |
| 24 | 7.77 | 1.34 | 4.52 | 411.5 | 0.75 | 3881 |
| Mean | 7.84 | 1.48 | 4.55 | 345.6 | 0.85 | 3413 |
| SD (%) | 0.43 | 5.37 | 5.29 | 144.1 | 6.51 | 8.95 |

Table H. Individual best-fit parameters – Simplified Model 1.

| **ID** | $\boldsymbol{-lo}\boldsymbol{g}_{\boldsymbol{10}}\left( \boldsymbol{\beta} \right)$ | $\boldsymbol{\lambda}_{\boldsymbol{E}}$ | $\boldsymbol{b}$ | $\boldsymbol{K}_{\boldsymbol{B}}$ | $\boldsymbol{m}$ | $\boldsymbol{p}$ | **covariate** |
| --- | --- | --- | --- | --- | --- | --- | --- |
| 1 | 7.82 | 0.54 | 5.23 | 0.94 | 1.69 | 3097 | 1 |
| 2 | 7.83 | 0.54 | 5.75 | 0.78 | 1.69 | 3179 | 1 |
| 3 | 7.8 | 0.48 | 5.09 | 2.17 | 1.69 | 3261 | 1 |
| 4 | 7.82 | 0.54 | 5.14 | 0.74 | 1.66 | 3112 | 1 |
| 5 | 7.8 | 0.48 | 5.29 | 2.89 | 1.7 | 3253 | 1 |
| 6 | 7.8 | 0.49 | 4.85 | 1.86 | 1.69 | 3276 | 1 |
| 7 | 7.79 | 0.47 | 5.38 | 2.74 | 1.68 | 3351 | 1 |
| 8 | 7.86 | 0.66 | 5.49 | 0.92 | 1.86 | 2722 | 1 |
| 9 | 7.82 | 0.55 | 5.67 | 0.64 | 1.71 | 3078 | 1 |
| 10 | 7.85 | 0.52 | 5.13 | 1488 | 1.72 | 2657 | 2 |
| 11 | 7.87 | 0.6 | 5.15 | 359.8 | 1.76 | 3123 | 2 |
| 12 | 7.86 | 0.55 | 4.25 | 878.9 | 1.72 | 2977 | 2 |
| 13 | 7.78 | 0.45 | 4.97 | 603.1 | 1.65 | 3424 | 2 |
| 14 | 7.85 | 0.58 | 5.35 | 160.7 | 1.74 | 3062 | 2 |
| 15 | 7.81 | 0.54 | 6.18 | 75.81 | 1.71 | 3104 | 2 |
| 16 | 7.77 | 0.47 | 5.78 | 80.96 | 1.66 | 3466 | 2 |
| 17 | 7.83 | 0.52 | 4.61 | 699.7 | 1.73 | 3066 | 2 |
| 18 | 7.78 | 0.51 | 6.41 | 20.21 | 1.75 | 3331 | 2 |
| 19 | 7.86 | 0.63 | 5.26 | 201.7 | 1.92 | 2764 | 2 |
| 20 | 7.79 | 0.51 | 6.24 | 55.49 | 1.7 | 3241 | 2 |
| 21 | 7.82 | 0.48 | 4.2 | 1104 | 1.7 | 3179 | 2 |
| 22 | 7.76 | 0.42 | 4.45 | 1125 | 1.6 | 3749 | 2 |
| 23 | 7.83 | 0.55 | 5.41 | 227.7 | 1.67 | 3106 | 2 |
| 24 | 7.76 | 0.43 | 5.24 | 455.6 | 1.63 | 3613 | 2 |
| Mean | 7.82 | 0.52 | 5.27 | 1.52/502.4 | 1.71 | 3175 |  |
| SD (%) | 0.41 | 11.07 | 10.57 | 56.04/89.14 | 3.84 | 7.85 |  |

Table I. Individual best-fit parameters – Simplified Model 1 with a covariate on $K_{B}$ (1 is PTC and 2 is NC). For $K_{B}$, the mean and SD (%) are reported for individual group PTC/NC.

| **ID** | $\boldsymbol{-lo}\boldsymbol{g}_{\boldsymbol{10}}\left( \boldsymbol{\beta} \right)$ | $\boldsymbol{\lambda}_{\boldsymbol{E}}$ | $\boldsymbol{b}$ | $\boldsymbol{K}_{\boldsymbol{B}}$ | $\boldsymbol{m}$ | $\boldsymbol{p}$ |
| --- | --- | --- | --- | --- | --- | --- |
| 1 | 7.86 | 1.89 | 8.06 | 21.76 | 0.6 | 2681 |
| 2 | 7.85 | 1.55 | 8.16 | 1.24 | 0.47 | 3088 |
| 3 | 7.83 | 1.31 | 7.94 | 3.81 | 0.42 | 3177 |
| 4 | 7.86 | 1.74 | 7.15 | 7.44 | 0.54 | 2603 |
| 5 | 7.83 | 1.28 | 8.1 | 5.15 | 0.44 | 3187 |
| 6 | 7.86 | 1.99 | 7.54 | 142.6 | 0.53 | 2648 |
| 7 | 7.81 | 1.18 | 7.7 | 4.1 | 0.41 | 3309 |
| 8 | 7.87 | 2.17 | 7.66 | 65.31 | 0.58 | 2694 |
| 9 | 7.9 | 2.25 | 7.12 | 37.28 | 0.61 | 3122 |
| 10 | 7.86 | 1.41 | 7.99 | 1950 | 0.41 | 2462 |
| 11 | 7.88 | 1.81 | 7.48 | 493.7 | 0.53 | 3127 |
| 12 | 7.86 | 1.52 | 6.93 | 1439 | 0.47 | 2788 |
| 13 | 7.8 | 1.1 | 7.62 | 971.2 | 0.39 | 3341 |
| 14 | 7.88 | 1.33 | 7.78 | 179.1 | 0.44 | 2955 |
| 15 | 7.83 | 1.34 | 7.66 | 64.83 | 0.44 | 3085 |
| 16 | 7.79 | 1.15 | 7.81 | 92.8 | 0.39 | 3406 |
| 17 | 7.85 | 1.44 | 7.56 | 1147 | 0.44 | 2975 |
| 18 | 7.81 | 1.21 | 7.81 | 19.49 | 0.41 | 3267 |
| 19 | 7.87 | 2.02 | 7.58 | 235.1 | 0.55 | 2762 |
| 20 | 7.82 | 1.2 | 7.7 | 58.35 | 0.41 | 3220 |
| 21 | 7.82 | 1.01 | 7.58 | 1586 | 0.41 | 3114 |
| 22 | 7.77 | 1.04 | 7.29 | 2004 | 0.39 | 3723 |
| 23 | 7.85 | 1.41 | 7.47 | 266.1 | 0.45 | 3046 |
| 24 | 7.77 | 1.08 | 7.67 | 655.7 | 0.39 | 3561 |
| Mean | 7.84 | 1.48 | 7.64 | 477.1 | 0.46 | 3056 |
| SD (%) | 0.43 | 24.59 | 3.94 | 135.8 | 15.01 | 10.03 |

Table J. Individual best-fit parameters – Simplified Model 2.

| **ID** | $\boldsymbol{-lo}\boldsymbol{g}_{\boldsymbol{10}}\left( \boldsymbol{\beta} \right)$ | $\boldsymbol{\lambda}_{\boldsymbol{E}}$ | $\boldsymbol{b}$ | $\boldsymbol{K}_{\boldsymbol{B}}$ | $\boldsymbol{m}$ | $\boldsymbol{d}$ | $\boldsymbol{K}_{\boldsymbol{D}}$ | $\boldsymbol{p}$ |
| --- | --- | --- | --- | --- | --- | --- | --- | --- |
| 1 | 7.66 | 8.54 | 7.61 | 40.57 | 0.31 | 0.042 | 100000 | 2542 |
| 2 | 7.65 | 8.62 | 7.67 | 3.52 | 0.28 | 0.038 | 100000 | 2665 |
| 3 | 7.64 | 8.22 | 7.22 | 10.19 | 0.28 | 0.04 | 100000 | 2744 |
| 4 | 7.67 | 8.66 | 7.58 | 19.98 | 0.31 | 0.038 | 100000 | 2600 |
| 5 | 7.64 | 8.14 | 7.6 | 9.38 | 0.26 | 0.04 | 100000 | 2920 |
| 6 | 7.67 | 8 | 6.91 | 246.8 | 0.31 | 0.048 | 100000 | 2504 |
| 7 | 7.64 | 7.92 | 7.41 | 8.13 | 0.26 | 0.044 | 100000 | 2986 |
| 8 | 7.65 | 8.43 | 6.95 | 112.5 | 0.29 | 0.043 | 100000 | 2299 |
| 9 | 7.67 | 8.56 | 7.18 | 75 | 0.32 | 0.032 | 100000 | 2582 |
| 10 | 7.64 | 8.12 | 7.1 | 6727 | 0.26 | 0.05 | 100000 | 2296 |
| 11 | 7.65 | 8.37 | 7.3 | 1230 | 0.29 | 0.043 | 100000 | 2624 |
| 12 | 7.65 | 8.21 | 6.35 | 4088 | 0.27 | 0.046 | 100000 | 2500 |
| 13 | 7.63 | 7.8 | 6.42 | 1586 | 0.26 | 0.044 | 100000 | 3049 |
| 14 | 7.65 | 8.25 | 6.2 | 398.3 | 0.28 | 0.044 | 100000 | 2625 |
| 15 | 7.64 | 8.05 | 6.8 | 122.4 | 0.27 | 0.044 | 100000 | 2815 |
| 16 | 7.63 | 7.83 | 7.18 | 161.7 | 0.25 | 0.045 | 100000 | 3148 |
| 17 | 7.64 | 8.15 | 6.24 | 2252 | 0.27 | 0.043 | 100000 | 2698 |
| 18 | 7.63 | 7.91 | 6.97 | 33.62 | 0.26 | 0.043 | 100000 | 3006 |
| 19 | 7.66 | 8.46 | 7.03 | 542.3 | 0.29 | 0.042 | 100000 | 2436 |
| 20 | 7.64 | 8 | 6.94 | 106 | 0.26 | 0.042 | 100000 | 2945 |
| 21 | 7.64 | 8.12 | 6.4 | 4174 | 0.27 | 0.042 | 100000 | 2859 |
| 22 | 7.63 | 7.6 | 5.9 | 3074 | 0.25 | 0.043 | 100000 | 3405 |
| 23 | 7.65 | 8.13 | 6.69 | 548.6 | 0.27 | 0.044 | 100000 | 2733 |
| 24 | 7.63 | 7.51 | 6.5 | 1034 | 0.25 | 0.044 | 100000 | 3259 |
| Mean | 7.65 | 8.15 | 6.92 | 1109 | 0.28 | 0.043 | 100000 | 2760 |
| SD (%) | 0.16 | 3.67 | 7.04 | 154.5 | 7.32 | 8.05 | 0 | 10.20 |

Table K. Individual best-fit parameters – Simplified Model 3.

| **ID** | $\boldsymbol{-lo}\boldsymbol{g}_{\boldsymbol{10}}\left( \boldsymbol{\beta} \right)$ | $\boldsymbol{m}$ | $\boldsymbol{p}$ |
| --- | --- | --- | --- |
| 1 | 7.69 | 1.44 | 2767 |
| 2 | 7.69 | 1.46 | 2744 |
| 3 | 7.69 | 1.45 | 2775 |
| 4 | 7.69 | 1.45 | 2759 |
| 5 | 7.69 | 1.45 | 2790 |
| 6 | 7.69 | 1.44 | 2775 |
| 7 | 7.69 | 1.44 | 2787 |
| 8 | 7.69 | 1.5 | 2669 |
| 9 | 7.69 | 1.45 | 2751 |
| 10 | 7.69 | 1.38 | 2855 |
| 11 | 7.68 | 1.38 | 2886 |
| 12 | 7.68 | 1.36 | 2901 |
| 13 | 7.68 | 1.33 | 2962 |
| 14 | 7.68 | 1.41 | 2853 |
| 15 | 7.68 | 1.37 | 2911 |
| 16 | 7.68 | 1.32 | 2951 |
| 17 | 7.68 | 1.35 | 2958 |
| 18 | 7.69 | 1.44 | 2836 |
| 19 | 7.69 | 1.44 | 2782 |
| 20 | 7.69 | 1.44 | 2841 |
| 21 | 7.68 | 1.32 | 3020 |
| 22 | 7.68 | 1.34 | 2958 |
| 23 | 7.68 | 1.37 | 2914 |
| 24 | 7.68 | 1.27 | 3148 |
| Mean | 7.69 | 1.4 | 2858 |
| SD (%) | 0.065 | 4.08 | 3.69 |

Table L. Individual best-fit parameters – Simplified Model 4.

| **ID** | $\boldsymbol{-lo}\boldsymbol{g}_{\boldsymbol{10}}\left( \boldsymbol{\beta} \right)$ | $\boldsymbol{m}$ | $\boldsymbol{p}$ |
| --- | --- | --- | --- |
| 1 | 7.84 | 1.21 | 3519 |
| 2 | 7.84 | 1.22 | 3496 |
| 3 | 7.84 | 1.21 | 3531 |
| 4 | 7.84 | 1.21 | 3512 |
| 5 | 7.84 | 1.21 | 3547 |
| 6 | 7.84 | 1.21 | 3529 |
| 7 | 7.84 | 1.21 | 3544 |
| 8 | 7.85 | 1.24 | 3414 |
| 9 | 7.84 | 1.22 | 3502 |
| 10 | 7.83 | 1.17 | 3607 |
| 11 | 7.83 | 1.17 | 3643 |
| 12 | 7.83 | 1.14 | 3643 |
| 13 | 7.82 | 1.13 | 3725 |
| 14 | 7.83 | 1.2 | 3612 |
| 15 | 7.81 | 1.14 | 3745 |
| 16 | 7.82 | 1.14 | 3687 |
| 17 | 7.82 | 1.14 | 3699 |
| 18 | 7.83 | 1.21 | 3554 |
| 19 | 7.84 | 1.21 | 3536 |
| 20 | 7.83 | 1.21 | 3571 |
| 21 | 7.81 | 1.13 | 3762 |
| 22 | 7.82 | 1.15 | 3706 |
| 23 | 7.82 | 1.16 | 3664 |
| 24 | 7.8 | 1.1 | 3861 |
| Mean | 7.83 | 1.18 | 3609 |
| SD (%) | 0.15 | 3.21 | 2.87 |

Table M. Individual best-fit parameters – Simplified Model 5.

**Section E. Analytical approximation of the early viral rebound dynamics**

In order to better explain why $K_{B}$ separates the NC and PTC participants, we approximate analytically the viral set point after rebound for Simplified Model 1:

$$\frac{dT}{dt}=\lambda_{T}-d_{T}T-\beta VT$$

$$\frac{dI}{dt}=\left( 1-f_{L} \right)\beta VT+aL-\delta I-mEI$$

$$\frac{dL}{dt}=f_{L}\beta VT-d_{L}L-aL+\rho L$$

$$\frac{dE}{dt}=\lambda_{E}+\frac{bEI}{K_{B}+I}-d_{E}E$$

$$V=\frac{p}{c}I.$$

We are interested in the viral set point attained $<1$ year post-ATI and as HIV-1 infection progresses slowly, we approximate the target cell population by its disease-free equilibrium$T=\lambda_{T}/d_{T}$. It is worth pointing out that PWH, who experienced rapid viral rebound and potentially rapid decline of target cells, re-started ART. This approximation also works for the period before ART.

In this deterministic model, the contribution from latent cells to the viral rebound dynamics (e.g., the rate that virus becomes detectable and the magnitude of the set point viral load) is negligible. Thus, we assume that $L=0$ for this approximation, which will simplify the algebraic details further. Together, these considerations result in the following transient system which approximates the early viral rebound dynamics of the full model:

$$I^{'}=\left( \left( 1-f_{L} \right)\beta\left( \frac{p}{c} \right)\left( \frac{\lambda_{T}}{d_{T}} \right)-\delta-mE \right)I$$

$$E^{'}=\lambda_{E}+\frac{bEI}{K_{B}+I}-d_{E}E.$$

This approximated system with one infected cell compartment and one effector cell compartment is inspired by a previous study (Baral et al., 2019). Now, solving for $E^{'}=0$ gives

$$E_{ss}=\frac{\lambda_{E}\left( K_{B}+I \right)}{d_{E}\left( K_{B}+I \right)-bI}.$$

Substituting this expression for $E$ into the $I'$ equation,

$$I^{'}=\left( \left( 1-f_{L} \right)\beta\left( \frac{p}{c} \right)\left( \frac{\lambda_{T}}{d_{T}} \right)-\delta-m\frac{\lambda_{E}\left( K_{B}+I \right)}{d_{E}\left( K_{B}+I \right)-bI} \right)I.$$

Setting $I^{'}=0$ and solving for the non-zero steady state $I_{ss}$ gives

$$I_{ss}=\frac{\left[ m\lambda_{E}-\left( \left( 1-f_{L} \right)\beta\left( \frac{p}{c} \right)\left( \frac{\lambda_{T}}{d_{T}} \right)-\delta\right)d_{E} \right]K_{B}}{\left( \left( 1-f_{L} \right)\beta\left( \frac{p}{c} \right)\left( \frac{\lambda_{T}}{d_{T}} \right)-\delta\right)\left( d_{E}-b \right)-m\lambda_{E}}.$$

The corresponding critical value $V_{c}$ for $V\left( t \right)$ is given by

$$V_{ss}=\frac{p}{c}I_{ss}=\frac{p}{c}\frac{\left[ m\lambda_{E}-\left( \left( 1-f_{L} \right)\beta\left( \frac{p}{c} \right)\left( \frac{\lambda_{T}}{d_{T}} \right)-\delta\right)d_{E} \right]K_{B}}{\left( \left( 1-f_{L} \right)\beta\left( \frac{p}{c} \right)\left( \frac{\lambda_{T}}{d_{T}} \right)-\delta\right)\left( d_{E}-b \right)-m\lambda_{E}}.$$

Fig K shows a comparison of $V_{ss}$ with the predicted viral load set-point obtained with the full model dynamics.

**
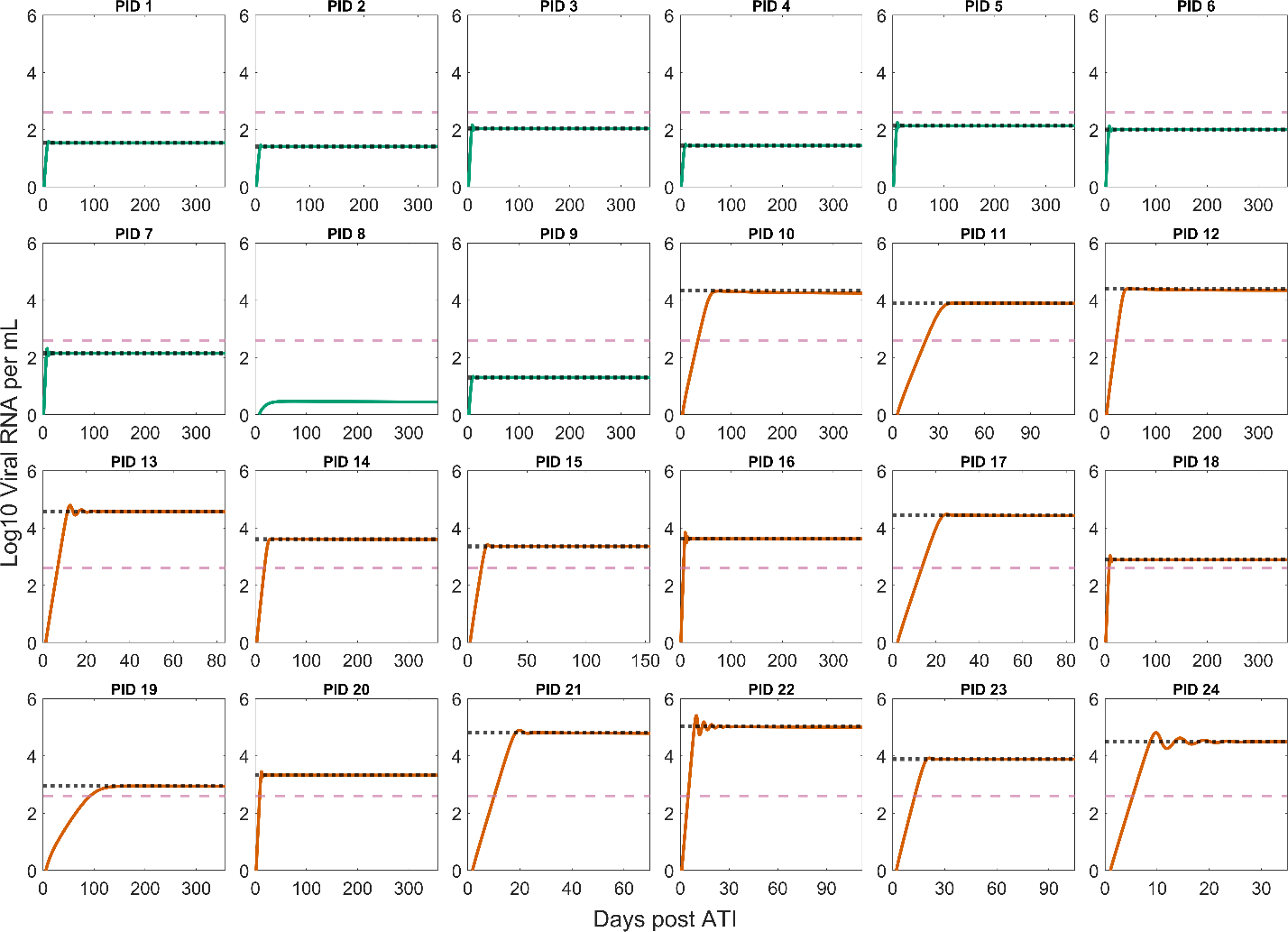
**

Fig K. Approximation of the set point viral load $V_{\mathrm{ss}}$ using the best fit parameters for each participant compared with the numerical solution of Simplified Model 1 (Eqn. 2). The horizontal black dotted line is the approximation$V_{\mathrm{ss}}$ (Eqn. 3). Green and dark orange curves correspond to the viral load of the PTC and NC participants, respectively, as predicted by the Simplified Model 1. The horizontal pink dashed line is the 400 viral RNA copies/mL threshold used in the classification of PTC. For participant number 8,$V_{\mathrm{ss}}$ is at 0.61 viral RNA copies/mL, which is the full model predicted viral set point after day ~12000.

To arrive at this approximation of the viral set point, we made several crude simplifications, where the assumptions of limited exhaustion$\left( \frac{dEI}{K_{D}+I}\approx0 \right)$ and constant target cells$\left( T=\frac{\lambda_{T}}{d_{T}} \right)$ resulted in the removal of one of the two non-extinction steady states of the full model. However, it is not clear whether or when both steady states are positive since the algebraic calculation is fairly complicated for the full model. The model also contains a disease-free steady state, which is unstable assuming the baseline immune response is not sufficient to clear the virus on its own.
