# Supplementary material for: Understanding early HIV-1 rebound dynamics following antiretroviral therapy interruption: The importance of effector cell expansion
Source: PLoS Pathog. 2024 Jul 29;20(7):e1012236. doi: 10.1371/journal.ppat.1012236 (PMC11309407; doi:10.1371/journal.ppat.1012236)
Supplement: Attachment — Submitted filename: Response to Reviewer Comments.docx [file ppat.1012236.s003.docx]

Response to Reviewer Comments

**Part I - Summary**
Please use this section to discuss strengths/weaknesses of study, novelty/significance, general execution and scholarship.

Reviewer #1: The manuscript is well-written with sufficient reference to the existing literature on HIV latency and the mathematical modeling thereof. The primary finding is that both the dynamics of viral rebound following the interruption of ART and the variation observed between post treatment controllers and noncontrollers are captured by a model with simplified effector cell dynamics (no exhaustion). Further, the distinction between PTC and NC can be made with a single parameter (K_B).

Models of viral infection that include immune cells come in many varieties, at many levels of complexity, and they all have many parameters. To my eyes, the significance of the results presented here are not necessarily in the specific mechanisms suggested (which will require much further experimental work to either confirm or reject) but the fact that a relatively simple model is able to capture the dynamics. As a result, future complex models for viral rebound will need to justify the level of complexity they contain or acknowledge that the dynamic of rebound are already explained by a simpler submodel.

The general execution of this manuscript is excellent, and I have only minor comments to add prior to its acceptance.

Also, the supplementary materials provided are extremely helpful.

Thank you for your positive feedback. We appreciate your recognition of our model's simplicity in capturing the dynamics of viral rebound and its potential impact on future research.

Reviewer #2: The paper studies the complex dynamics of HIV-1 rebound post-ART interruption. The authors developed a dynamic model based on the theoretical framework proposed by Conway and Perelson, which considers virus-immune interactions to understand viral rebound dynamics. The model is evaluated using viral load data from 24 individuals, highlighting the significance of the effector cell expansion rate in distinguishing post-treatment controllers (PTC) from non-controllers (NC).

Thank you for your thoughtful comments of our work.

**Part II – Major Issues: Key Experiments Required for Acceptance**
Please use this section to detail the key new experiments or modifications of existing experiments that should be absolutely required to validate study conclusions.

Generally, there should be no more than 3 such required experiments or major modifications for a "Major Revision" recommendation. If more than 3 experiments are necessary to validate the study conclusions, then you are encouraged to recommend "Reject".

Reviewer #1: N/A

Reviewer #2: I do not find major issues.

**Part III – Minor Issues: Editorial and Data Presentation Modifications**
Please use this section for editorial suggestions as well as relatively minor modifications of existing data that would enhance clarity.

Reviewer #1: In describing the original Conway & Perelson model (l.98-l.124) care is sometimes taken about whether rates are constant or per capita constant, but at other times the distinction is not made. For example, d_T should be per capita, while it is unclear what the proper label for m would be. These discrepancies should be rectified, or should the could simply be referred to as "rates".

We have added clarifications for these rates. In particular, *m* is a second order reaction rate constant, which is used to describe the reaction of two reactants, i.e. E and I. We added this as a footnote to clarify what we meant by calling *m* a rate constant. In Table 1 we now call *m* the effector cell killing rate constant.

When discussing data fitting (l.163-l.178) it is slightly confusing to read references to parameters, such as K_D, that do not appear in the main model.

We have added a clarification that $K_{D}$ appears in other models that we examined.

In Figure 1, the x-axes should all be the same. It is good that the existence of different axes is mentioned in the text, but I do not feel that the gain in ease of seeing individual data points outweighs the loss in visibility of dynamic rates.

We initially used the same x-axis for all participants; however, due to several participants having rapid rebound (such as 781051) and were put back on ART, it can be difficult to see the data points and the fitting for these individuals. We include here Fig 1 with the same x-axis for all participants to demonstrate our point. We also plotted the data points smaller and used lighter colors to differentiate the fitting curves and the data points to improve the visibility. However, for several participants, the overall visibility is still poor. Thus, we prefer to keep Fig 1 as in our original submission.

Relatedly, the reason that some patients datasets are truncated (resumption of ART) is mentioned only in the caption of Figure 1, and should be mentioned in the primary text.

We have added this clarification in the Data part of the Methods.

In the supplementary materials, the BICc for Simplified Model 2 is very close to that of Simplified Model 1. It seems worth a mention in the main text that fits of similar quality are achieve with a model that includes no dynamics in the latent cell class.

We have added a note to the discussion regarding this point. It is likely that due to the maximum one-year constraint on the data and the slow dynamics of the latently infected cells, its impact cannot be observed. This may be similar to why the exhaustion effect is likely unobservable with this data set.

Reviewer #2: The study is based on data from 24 individuals, which may not be representative of the broader population of people living with HIV-1. The authors may want to add this limitation to the discussion section.

We have added clarification regarding this limitation in the discussion section.

While the model finds limited impact of effector cell exhaustion in the short term, it does not fully explore its potential role in long-term viral dynamics and control. If these PTCs experience viral rebound later, does that mean effector cell exhaustion begins to play a role?

We have elaborated on this point further in the discussion. Further analysis will need to use longer longitudinal data.

On page 4, lines 122-123, the authors mention homeostatic proliferation of latently infected cells. However, Ref 68 concerns asymmetric cell division. The following references discuss latency homeostasis: (1) PLoS Computational Biology 5 (10), e1000533, 2009; (2) Journal of Theoretical Biology 260 (2), 308-331, 2009.

We have added these references that contain previous formulations of latent cell homeostasis.
